# Editorial: Advances and insights in the diagnosis of viral infections and vaccines development in animals

**DOI:** 10.3389/fmicb.2024.1443858

**Published:** 2024-07-02

**Authors:** Lihua Wang, Jingqiang Ren, Jianke Wang, Hewei Zhang, Jishu Shi

**Affiliations:** ^1^Center on Biologics Development and Evaluation, Department of Anatomy and Physiology, College of Veterinary Medicine, Kansas State University, Manhattan, KS, United States; ^2^Institute of Virology, Wenzhou University, Wenzhou, China; ^3^Hebei Veterinary Biotechnology Innovation Center, College of Veterinary Medicine, Hebei Agricultural University, Baoding, China; ^4^College of Food and Drugs, Luoyang Polytechnic, Luoyang, Henan, China

**Keywords:** advances, insights, diagnosis, vaccine, viral infection, animal

## Introduction

Animal health is vital to global wellbeing, economic development, food security, and food quality. However, animal viruses pose a significant threat, causing livestock and wildlife illnesses, economic hardship, and zoonotic diseases that can cross species barriers to endanger human health. Severe acute respiratory syndrome (SARS), avian influenza A (H5N1), and Coronavirus disease 2019 (COVID-19) serve as stark reminders of these risks (Yuen et al., 1998; Peiris et al., 2003; Zhu et al., 2020). To combat these viral foes, robust strategies are urgently needed. Accurate diagnoses of viral infections and the development of effective vaccines are critical components of such strategies. This Research Topic, “*Advances and insights in the diagnosis of viral infections and vaccines development in animals*,” covered the development of novel diagnostic tools for various animal viruses and explored advancements in vaccine development utilizing diverse technologies. The Research Topic comprised 23 articles, with 11 focusing on the development, evaluation, and application of diagnostic methods. The remaining 12 articles emphasized vaccine development and evaluation.

## Diagnosis of viral infections in animals

Traditionally, diagnosing viral infections in animals relied on clinical signs, pathology, antigen detection, and antibody detection. Clinical signs and pathological findings in viral infected animals are highly variable due to both viral and host factors, and often confused with other diseases of animals (Murcia et al., 2009; Wang et al., 2020). Well-established laboratory techniques like virus isolation, real-time polymerase chain reaction (RT-PCR), and enzyme-linked immunosorbent assay (ELISA) have improved diagnostic accuracy (Wang et al., 2020, 2022; Mi et al., 2022; Azam et al., 2023). Recent advancements have taken this progress a step further by offering faster, more efficient methods. In this Research Topic, Yu et al. developed a colloidal gold immunochromatographic (GICG) strip with an enhanced signal according to the double-antibody sandwich principle and an enzyme-based signal amplification system to amplify the signal for detecting bovine parvovirus (BPV). The sensitivity of the signal-enhanced GICG strip showed 10 times higher than that of the traditional GICG strip. Liu, Sheng et al. developed a sensitive and specific TaqMan-based quantitative real-time PCR assay to detect the novel Mink Circovirus (MiCV). Li, Liu et al. generated a monoclonal antibody against duck circovirus capsid protein and investigated its potential application for native viral antigen detection. Li, Wang et al. developed propidium monoazide quantitative PCR assay for effective discrimination of infectious and inactivated African swine fever virus (ASFV). Its integration into routine diagnostics can significantly enhance the interpretation of positive ASFV results, leading to improved accuracy in identifying infected cases.

Multiplex PCR assays significantly boost diagnostic productivity by simultaneously detecting multiple pathogens from a single reaction. This approach offers several advantages: reduced costs, minimal sample requirement, and faster turnaround times (Shi et al., 2016; Dronina et al., 2021; Charlier et al., 2022). Ren, Zu et al. developed a multiplex real-time PCR assay using TaqMan probes to simultaneously detect Porcine epidemic diarrhea virus (PEDV), porcine rotavirus (PoRV), and porcine deltacoronavirus (PDCoV), the important diarrhea viruses in pig herds. This method is expected to significantly contribute to prevent and control the spread of infectious diseases, as well as aid in conducting epidemiological investigations. Yan et al. designed a dual nanoparticle-assisted polymerase chain reaction (Nano-PCR) assay for simultaneous detection of Feline calicivirus (FCV) and Feline herpesvirus type I (FHV-I). The assay showed strong specificity and high sensitivity for testing the clinical samples of feline upper respiratory tract infections.

Next-generation sequencing (NGS) has revolutionized viral diagnosis. It allows identification of previously unknown or uncultivable viruses, a significant advancement over traditional methods (Quer et al., 2022). In this Research Topic, Goraichuk et al. developed a superior method with DNase to deplete host ribosomal RNA (rRNA) before library preparation. This method significantly improves the sensitivity and accuracy of virus detection in clinical samples using NGS. Their 28S rRNA RT-qPCR assay provided a valuable foundation for the development of these host depletion strategies. Additionally, machine learning, powered by modern computing, has emerged as a powerful tool for data analysis and disease diagnosis. Liu, Zhou et al. combined machine learning with causal relationship analysis to identify shared mechanisms between COVID-19 and acute myocardial infarction (AMI). They utilized 20 mainstream machine learning algorithms to establish a powerful diagnostic predictor. This tool can estimate a specific COVID-19 patient's risk of developing AMI. These findings offer novel mechanistic insights into COVID-19 and AMI, paving the way for future advancements in preventive, personalized, and precision medicine.

Several studies of this Research Topic highlight the application of diagnostics in animal health. Sánchez-Morales et al. used a surrogate ELISA kit to analyze blood serum from randomly selected animals in a retrospective study of SARS-CoV-2. Their findings suggest a higher susceptibility to infection in cats compared to dogs. Additionally, the study revealed a significantly increased infection risk for domestic animals living in close contact with infected owners, compared to those in animal shelters with limited human interaction. Uddin et al. employed insulated isothermal PCR (iiPCR) alongside traditional PCR and virus isolation to detect Lumpy skin disease virus (LSDV) in tissue samples from affected cattle. This approach provided insights into the potential source of the circulating LSDV strain and identified the inactivated LSDV antigen as a promising vaccine candidate. Magouz et al. investigated the prevalence of canine parvovirus-2 (CPV-2) variants among dogs in Egypt. Their study utilized PCR and restriction fragment length polymorphism (RFLP) followed by VP2 sequencing on samples from clinically infected dogs. The results confirmed the widespread presence of CPV-2 in the Egyptian canine population, emphasizing the need for continuous monitoring. The data can facilitate early and accurate diagnosis of the disease, ultimately aiding in the development of new vaccination strategies for Egypt.

## Vaccine development in animals

Given the absence of broad-spectrum antiviral pharmaceuticals, vaccination remains a critical tool for preventing and controlling viral infections in animals (McVey and Shi, 2010; Choudhury et al., 2021). Animal vaccines not only combat diseases in companion animals but also ensure the safety of food supplies by maintaining healthy livestock populations. Depending on the types of technologies used for antigen production and vaccine formulation, there are four types of vaccines for animal use: (i) Type I (whole virus): inactivated, killed, (ii) Type II (whole virus): modified live attenuated, reverse genetics modified, (iii) Type III (fraction/component): subunit, virus like particles, genetic DNA or RNA, killed recommitment vectors, (iv) Type IV (fraction/component): recombinant viral vectors expressing antigens (Brun, 2016). Numerous conventional Type I and Type II vaccines have been produced for companion and livestock (Coetzee et al., 2020; Lu et al., 2022; Yuan et al., 2022; Natesan et al., 2023). This Research Topic highlights advancements in Type I and Type II vaccine development. Shin et al. demonstrated that foot-and-mouth disease (FMD) inactivated vaccine with a glycyrrhizic acid adjuvant elicits potent innate and adaptive immune responses against FMD in mice and pigs. Cao et al. employed a yeast-based transformation-associated recombination (TAR) system to genetically engineer feline infectious peritonitis virus for vaccine development. Xu et al. successfully generated promising live attenuated pseudorabies virus (PRV) vaccine candidates using a codon deoptimization approach. One candidate exhibited good safety and a high level of virus neutralization in piglets, making it a potential solution against PRV variants.

An increasing number of rationally designed Type III and Type IV vaccines are developed and reaching the market (Madera et al., 2018; Shi et al., 2021; Tabynov et al., 2022). This Research Topic showcases seven articles exploring advancements in these vaccine types. Zhang Y. et al. investigated *Bacillus subtilis* (*B. subtilis*, a gram-positive bacterium that is safe and non-toxic to humans and animals) as a vector for developing oral rabies vaccine. They constructed recombinant *B. subtilis* expressing rabies virus G proteins. Their results suggested that recombinant *B. subtilis* strains have excellent immunogenicity and are expected to be novel oral vaccine candidates for the prevention and control of wild animal rabies. Ren, Madera et al. proposed a streamlined method for isolating high-quality viral DNA from large DNA viruses and generating recombinant PRV vaccines, simplifying vaccine development. Zhang H. et al. developed a yeast-based vaccine expressing influenza hemagglutinin (HA) proteins from H5N8, H7N9, and H9N2 strains. This oral vaccine in chickens showed promise in boosting multi-systemic immune responses against H9N2 influenza. Their finding suggest that oral yeast based multivalent bird flu vaccines provide an attractive strategy to update host defense function via reshapes of multi-systemic immune homeostasis. Zhao et al. identified the PRV gD protein as a potential vaccine candidate. Their findings suggest it could be effective in protecting animals from PRV infection. Hou et al. designed a multi-epitope vaccine targeting both porcine epidemic diarrhea virus (PEDV) and porcine deltacoronavirus (PDCoV). Zhou et al. identified a B-cell epitope in Senecavirus A (SVA) that could be used to develop a marker vaccine for the disease. Fan et al. identified L11L and L7L genes in the ASFV genome as virulence factors. Deleting these genes attenuated the virus, suggesting potential targets for future vaccines.

This Research Topic also emphasizes the importance of evaluating vaccine effectiveness against circulating viruses. Mosad et al. investigated genetic diversity between Avian Orthoreovirus (ARV) strains circulating in Egypt and the current vaccine strain. They found significant genetic and protein variation, suggesting the need for a new vaccine formulated from locally isolated ARV strains. Similarly, Yang et al. studied cross-protection among different feline calicivirus genotypes. Using cross-neutralization assays and *in vivo* challenges, they identified the DL39 strain as a promising candidate with broad-spectrum protection against various FCV genotypes.

## Conclusion and future perspectives

This Research Topic delves into the latest advancements in diagnosing and preventing viral infections in animals. It showcases a range of diagnostic tools, from established techniques like ELISA and PCR to cutting-edge methods like Nano-PCR, NGS, and machine learning. Vaccine development also takes center stage, with the exploration of both traditional inactivated vaccines and advanced subunit and recombinant vectored approaches. These advancements empower veterinarians and public health officials to safeguard animal and human health.

Looking ahead, the fight against animal viruses is an ongoing battle. Continuous improvement of diagnostic tools remains crucial. The future holds promise for on-site testing delivering rapid results, potentially transforming animal healthcare with faster and more targeted interventions. Similarly, research into universal vaccines with broad-spectrum protection offers immense potential, especially in the face of emerging viruses and antigenic variation. Enhancing international collaboration and information sharing can significantly accelerate vaccine development. Furthermore, improved pathogen detection in wildlife is a pressing need. We require robust tools for diagnosing emerging and re-emerging diseases that threaten endangered species or pose zoonotic risks. This includes developing innovative, non-invasive sampling methods, leveraging new technologies for comprehensive pathogen characterization, and refining data analysis and surveillance strategies (Jia et al., 2020). By staying at the forefront of scientific advancements and fostering continuous innovation, we can significantly improve the health of both animals and humans.

## Author contributions

LW: Writing – original draft, Writing – review & editing. JR: Writing – review & editing. JW: Writing – review & editing. HZ: Writing – review & editing. JS: Writing – review & editing.
